# Diagnosis of alcoholism based on neural network analysis of phenotypic risk factors

**DOI:** 10.1186/1471-2156-6-S1-S131

**Published:** 2005-12-30

**Authors:** Catherine T Falk

**Affiliations:** 1 CCNY of the City University of New York, 138^th ^Street and Convent Avenue, New York, NY 10031 USA

## Abstract

**Background:**

Alcoholism is a serious public health problem. It has both genetic and environmental causes. In an effort to gain understanding of the underlying genetic susceptibility to alcoholism, a long-term study has been undertaken. The Collaborative Study on the Genetics of Alcoholism (COGA) provides a rich source of genetic and phenotypic data. One ongoing problem is the difficulty of reliably diagnosing alcoholism, despite many known risk factors and measurements. We have applied a well known pattern-matching method, neural network analysis, to phenotypic data provided to participants in Genetic Analysis Workshop 14 by COGA. The aim is to train the network to recognize complex phenotypic patterns that are characteristic of those with alcoholism as well as those who are free of symptoms. Our results indicate that this approach may be helpful in the diagnosis of alcoholism.

**Results:**

Training and testing of input/output pairs of risk factors by means of a "feed-forward back-propagation" neural network resulted in reliability of about 94% in predicting the presence or absence of alcoholism based on 36 input phenotypic risk factors. Pruning the neural network to remove relatively uninformative factors resulted in a reduced network of 14 input factors that was still 95% reliable. Some of the factors selected by the pruning steps have been identified as traits that show either linkage or association to potential candidate regions.

**Conclusion:**

The complex, multivariate picture formed by known risk factors for alcoholism can be incorporated into a neural network analysis that reliably predicts the presence or absence of alcoholism about 94–95% of the time. Several characteristics that were identified by a pruned neural network have previously been shown to be important in this disease based on more traditional linkage and association studies. Neural networks therefore provide one less traditional approach to both identifying alcoholic individuals and determining the most informative risk factors.

## Background

Alcoholism, like many other complex traits, offers a challenge to those trying to categorize individuals as either normal or affected. If we are to succeed in finding genes underlying susceptibility to the trait or traits, it is necessary to have reliable methods for assigning disease phenotypes. Many diagnostic methods for alcoholism have been proposed that use a combination of responses on questionnaires, physical measurements, and observational data. Two of the main methods in use today are known as DSM-III-R+Feighner and DSM-IV-R. Results from these diagnosis standards are available for most individuals in the Collaborative Study on the Genetics of Alcoholism (COGA) database.

Information that was provided to participants of Genetic Analysis Workshop 14 (GAW14) included both diagnoses, together with a large number of "phenotypic" variables for each individual. Taken together, these variables provide a complex, multivariate picture of some of the information used in determining a diagnosis of alcoholism. In order to test the reliability of the given set of risk factors to predict the affection status of individuals in the dataset, we decided to apply a well known pattern matching technique to the data. We developed a back-propagation, feed-forward neural network using most of the risk factor data available. The risk factors were coded to provide input used to train the network to predict whether each individual's pattern of input factors indicated the presence of alcoholism or whether the pattern suggested a normal phenotype. We used the second diagnostic method mentioned above, namely DSM-IV-R, coded as ALDX2 in the GAW dataset. Our results indicate that neural networks can be useful in helping to determine the disease classification of individuals with respect to alcoholism.

## Methods

All individuals were coded for presence or absence of alcoholism as well as for risk factors as defined in Table 1. Individuals with missing data were not included in the analysis. Information on family relationships was not used. Following coding and culling, 650 records remained. These were then prepared for analysis by a neural network. There were 36 input values, all of which were either binary, or normalized to values between zero and 1. There was one output value: individuals with ALDX2 codes of 1 or 3 were coded as normal (0) and those with ALDX2 code 5 were considered affected (1). We should point out that the ALDX2 code of 1 indicated a "pure unaffected" individual and the code of 3 indicated an individual who was "unaffected with some symptoms". The computer program NNDRIVER [1] was used to train and validate neural networks, based on the input data. NNDRIVER employees a back-propagation, feed-forward structure, with one hidden layer and a single, binary outcome. The neural network was constructed with six nodes in the hidden layer. Initially all 36 input values were included. Neural networks were trained using a randomly selected set of 300 individuals. Following training, the network was validated using the remaining 350 individuals. This procedure was repeated three times to reduce the effect of randomly selecting a training set with special characteristics. Following the three replicate runs, the average scores for all individuals were calculated and compared to the output diagnoses supplied with the dataset.

After determining that training in the separate replicates was quite reliable (97–98.6%) and that validation was also quite good (85.7–90%), we attempted to determine which of the input parameters were most informative in obtaining a reliably trained neural network. To accomplish this we systematically pruned input factors and compared the results to those of the "full" neural network. Pruning was done by sequentially dropping one input factor at a time and noting the new number of "incorrect" predictions. Those input factors that had the most impact (i.e., increased the number of errors by more than 40% of the number of incorrect predictions for the entire set of input factors) were retained for the pruned network. The other input factors were dropped. Based on this pruning method, we selected 14 of the original 36 input factors and used them for training a streamlined neural network. We also examined the average differences between input values for the correctly and incorrectly classified individuals in the affected and unaffected classes to determine which parameters differed most significantly between the two groups.

## Results

**Figure 1:**
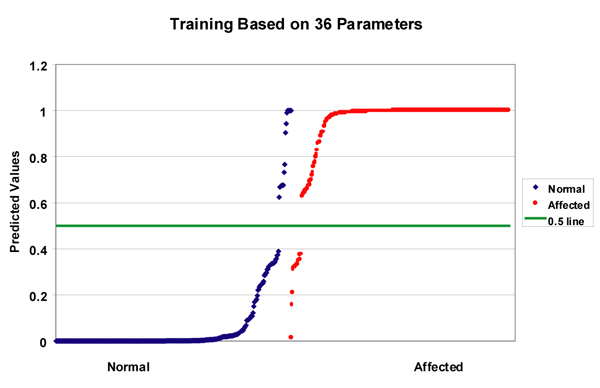
**Predicted values and true diagnoses for 650 individuals: full neural network**. Individuals diagnosed as normal are shown with blue diamonds, those diagnosed as alcoholics are shown with red circles. The best predictions are represented by values close to zero for normals and close to one for affected individuals.

**Figure 2:**
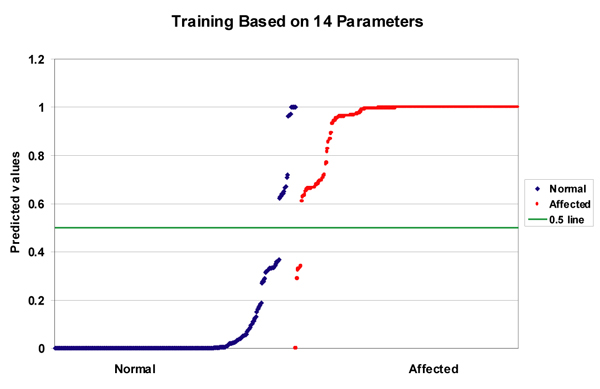
**Predicted values and true diagnoses for 650 individuals: pruned neural network**. Individuals diagnosed as normal are shown with blue diamonds, those diagnosed as alcoholics are shown with red circles. The best predictions are represented by values close to zero for normals and close to one fore affected individuals.

There were 650 records that fit the criteria for analysis. Of these, 44 were "pure unaffected" (code 1) for ALDX2, 293 were "unaffected with some symptoms" (code 3) and 313 were "affected" (code 5). For this analysis, the first two groups were coded as "normal" (0) and the last group was coded as "affected" (1). The initial run, using all 36 input factors, resulted in an average of 94% of the individuals being correctly classified. In this context "correctly classified" means that those coded 0 had a predicted value between 0 and 0.5 and those coded 1 had a predicted value between 0.5 and 1.0. However, values close to 0.5 cannot be considered reliable predictors. Eight of the 650 individuals (1.2%) fell into a "gray zone" between 0.4 and 0.6. Figure 1 shows predicted values and true diagnoses for all other individuals. Of these, 33 (5%) were incorrectly classified. All of the 44 "pure unaffected" individuals were correctly assigned (all with values close to zero). Eighteen "code 3" individuals were incorrectly classified along with 15 "code 5" individuals. A comparison of those correctly and incorrectly classified within each of those two groups showed that the major differences occurred with some of the 11 latent class variables and with the maximum number of drinks in one day. Many of the 14 phenotypic variables were very similar for both groups in the correct and incorrect classes. Similarly, factors such as age, ethnicity, and smoking habits did not differ, on average, between the correctly and incorrectly classified groups. After implementing the pruning method outlined above, 14 input factors were selected for testing with a streamlined neural network. These included: number of drinks per day (3 variables), latent class variables 1, 5, 7, 8, 9, and 11 (as defined in Table 1) and the phenotypic variables ttth1, ntth2, ntth3, ntth4, and ecb21. Training and validation of the pruned neural network gave results that were at least as good as the full network (96–98% for training, 84–90% for validation and 95% correctly classified). Figure 2 shows the predicted values and true diagnoses following the same training and validating procedure outlined above. In this case 19 (2.9%) were in the gray zone and are not shown here. Of the remaining individuals, 32 (5%) were misclassified: 9 affected individuals were classified as normal and 23 normal individuals were classified as affected. Again, all of the "pure unaffected individuals" were correctly classified.

## Discussion

Diagnosis of alcoholism is clearly a complex task, and several methods of classification have been devised to help determine a reliable, robust diagnosis. We have taken the set of risk factors and phenotypic measurements provided to GAW participants and have trained a neural network to classify individuals as either affected or normal. The factors appear to allow for fairly accurate training, with at least 97% agreement between the provided diagnosis and the predicted diagnosis in the full neural network. Validation, while not as high, is still quite good (between 85 and 90%). It is even possible to define a fairly narrow set of factors that continue to do a good job of predicting. It is interesting to note that several of the factors remaining in the pruned set have been cited in previous analyses of the COGA data as being linked to or associated with genes of interest. For example, ecb21 has been shown to exhibit linkage disequilibrium with GABA_A _receptor genes on chromosome 4 [2]. "Maximum number of drinks in a 24-hour period", when used as a quantitative trait, has also shown evidence of linkage on chromosome 4 near the alcohol dehydrogenase gene cluster [3]. The factor ttth1, also present in the reduced set of factors, has shown evidence of linkage on chromosome 7 [4]. Thus, the pruning may have identified several risk factors that are, in fact, likely to be linked to or associated with genes implicated in alcoholism.

It appears that one of the major factors important in separating correct from incorrect classification is the maximum number of drinks per day. Table 2 shows the average numbers for two of the ALDX2 classes, separated according to the neural network outcome. The average number of maximum drinks in one day reported by the "unaffected individuals with some symptoms" but classified by the neural network as affected is about 40% greater than the average reported by correctly classified "unaffected individuals with some symptoms". A similar decrease is seen in the maximum number of drinks reported by incorrectly classified affected individuals. Many of the discordant predictions are associated with individuals in the category where they are considered unaffected, "but with some symptoms". Based on the results of the neural network predictions, these individuals may be the most interesting to study in more detail. Perhaps the complexity of the neural network design is able to make more subtle distinctions between individuals in this category than is possible by more classical regression methods.

Neural network analysis does not necessarily replace the more standard regression methods. Rather, it may provide new (alternative) insight into the importance of risk factors (as in the case of individuals designated unaffected but with some symptoms. It would be interesting to compare the set of significant covariates identified by a regression analysis with the pruned set of risk factors in the neural network analysis. It would be encouraging to find significant overlap. The fact that several of the pruned factors have previously been identified in linkage or association studies suggests that this might be the case.

## Conclusion

The complex, multivariate picture formed by known risk factors for alcoholism can be incorporated into a neural network analysis that reliable predicts presence or absence of alcoholism about 94–95% of the time. Results show that one of the important indicators of susceptibility to alcoholism is the maximum number of drinks consumed in 24 hours. This characteristic and others that were identified by the pruned neural network have been shown to be important in this disease based on more traditional linkage and association studies. Neural networks therefore provide one less traditional approach to both identifying alcoholic individuals and determining the most informative risk factors.

## Abbreviations

COGA: Collaborative Study on the Genetics of Alcoholism

GAW14: Genetic Analysis Workshop 14

## Figures and Tables

**Table 1:** Coding of 36 risk factors for alcoholism

	**Category **	**Coded 0**	**Coded 1**	
**No.**	**Binary factors**			

	Sex	female	male	
	Smoker	no	yes	
				
1	Persistent desire to stop drinking	no	yes	
2	Morning drinking	no	yes	
3	Craving	no	yes	
4	Ever binge drink	no	yes	
5	So much time drinking...	no or < 1 month	yes > 1 month	
6	Narrowing of drinking repertoire	no	yes	
7	Gave up activities to drink	no	yes	
8	Blackouts (3 or more)	no	yes	
9	Withdrawal symptoms	no	yes	
10	Physical health problems	no	yes	
11	Emotional/psychological problems	no	yes	

	**Electrophysiological phenotypes**	Scaled from 0 to 1		

	**3-column binary coding**			

	Race	White	Black	other
	Drinks per day	< 5	5–10	> 10
	Age	< 20 years	20–40 years	> 40 years

**Table 2:** Comparison of maximum number of drinks in a 24-hour period between correctly and incorrectly classified individuals

ALDX2 class	Neural network outcome	Max. no. of drinks
Unaffected, some symptoms	Incorrect classification	23.12
	Correct classification	13.40

Affected	Incorrect classification	18.60
	Correct classification	29.58
